# Vonoprazan in erosive esophagitis: Influence of gender and hiatus hernia on outcomes

**DOI:** 10.12669/pjms.42.6.13929

**Published:** 2026-06

**Authors:** Muhammad Shoaib Khan, Mohammad Iltaf, Naeem Jan, Adnan ur Rehman

**Affiliations:** 1 Muhammad Shoaib Khan, FCPS(Gastro), Department of Gastroenterology, Hayatabad Medical Complex, Peshawar, Pakistan; 2 Mohammad Iltaf, FCPS (Gastro), Department of Gastroenterology, Hayatabad Medical Complex, Peshawar, Pakistan; 3 Naeem Jan, FCPS (Gastro), Department of Gastroenterology, Hayatabad Medical Complex, Peshawar, Pakistan; 4 Adnan ur Rehman, FCPS (Gastro), Department of Gastroenterology, Hayatabad Medical Complex, Peshawar, Pakistan

**Keywords:** Erosive esophagitis, Mucosal healing, Hiatus hernia, Vonoprazan

## Abstract

**Objective:**

This study aimed to determine the efficacy of vonoprazan in erosive esophagitis and association of gender and hiatus hernia with treatment outcomes.

**Methodology:**

This prospective single arm cohort study (before and after treatment) was conducted from 1^st^ January to 31st August 2025 in Department of Gastroenterology, HMC Peshawar. Non-probability convenience sampling technique was used. Total 220 patients with endoscopically confirmed erosive esophagitis (EE) and fulfilling inclusion criteria were included. Exclusion criteria included peptic ulcer disease, malignancy, prior gastrointestinal surgery, pregnancy and recent NSAID use. All patients received vonoprazan 20-mg once daily for Eight weeks. Demographic and clinical data were documented. Endoscopic evaluation was repeated post-treatment. Data was analyzed using descriptive statistics, with p < 0.05 considered statistically significant.

**Results:**

The mean age was 42.51±8.19 years, with 120 (54.5%) male and 100 (45.5%) female. Before treatment, 115(52.3%) had LA-C and 26(11.8%) had LA-D esophagitis. Following treatment, 191(86.8%) out of 220 patients had no esophagitis, 18(8.2%) had LA-A and 11(5%) had LA-B esophagitis (OR:6.59, 95%CI 4.45 - 9.73, p < 0.001). Complete healing rates were similar in male and female patients (p = 0.94). Among 58 patients having hiatus hernia, 35(60.34%) had no esophagitis post-treatment as compared to those without hernia where 156 out of 162 (96.29%) had no esophagitis post-treatment (OR:17.08, CI (6.47- 45.08), p < 0.001).

**Conclusion:**

Vonoprazan demonstrated high efficacy in healing erosive esophagitis, especially in patients without hiatus hernia while having similar efficacy in both genders. These findings highlight vonoprazan’s therapeutic potential in management of erosive esophagitis.

## INTRODUCTION

Erosive esophagitis (EE), a severe manifestation of gastroesophageal reflux disease (GERD), is characterized by visible mucosal breaks endoscopically and is graded by Los Angeles (LA) classification system.^1^ Despite the widespread use of proton pump inhibitors (PPIs) for its management, their limitations include delayed onset, short duration of action, and variability due to CYP2C19 metabolism, resulting in incomplete mucosal healing, especially in moderate-severe cases.^2^ Vonoprazan, a potassium-competitive acid blocker (P-CAB), has emerged as a potent acid suppressant offering faster and sustained acid inhibition than PPIs.^3^

Recent RCTs have shown Vonoprazan to be more effective than lansoprazole in EE, particularly in patients with LA-C/D esophagitis.^1^,^4^ Meta-analyses support Vonoprazan’s superiority in healing and maintaining remission in EE.^5^,^6^ Furthermore, vonoprazan has demonstrated efficacy as maintenance therapy, with lower relapses over 24 weeks compared to lansoprazole.^7^,^8^

Pharmacokinetics of vonoprazan enables more consistent pH control, maintaining intragastric pH >4 for >20 hours daily.^9^,^10^ This translates to rapid symptom relief and enhanced mucosal healing. Real-world and multicenter studies further confirms it across diverse patient populations.^11^,^12^

However, clinical outcomes may be influenced by factors such as gender and anatomical abnormalities like hiatus hernia, which may delay or allow incomplete healing, making anatomical evaluation crucial. Moreover, emerging data indicate that presence of motility disorders, such as achalasia, may further complicate healing in EE.^13^

Given these considerations and the limited data availability about effect of vonoprazan in above scenarios in Pakistan, this study aims to assess treatment response to vonoprazan in patients with EE and the impact of gender and hiatus hernia on mucosal healing.

## METHODOLOGY

This prospective single arm cohort study (before and after treatment) was conducted at Department of Gastroenterology, HMC Peshawar from 1^st^ January 2025 to 31^st^ August 2025. Primary objective was assessing healing of erosive esophagitis (EE) before and after treatment with vonoprazan 20mg orally once daily for eight weeks. Secondary objectives were assessing effects of gender and hiatus hernia on healing of EE. A non-probability convenience sampling technique was employed to include a total of 220 adult patients from the outpatient and endoscopy departments who met the inclusion criteria. This sample size was calculated after keeping power of study at 80% with 95% confidence level and expected healing proportion of about 85% based on previous published literature and keeping 10% dropout rate.

Eligible patients were between 18 and 65 years with a diagnosis of EE based on upper gastrointestinal (GI) endoscopy and graded using the Los Angeles (LA) classification (Grades A-D). Patients were excluded if they had undergone previous upper gastrointestinal surgery, history of malignancy, were pregnant or lactating, had peptic ulcer disease or were unable to attend follow-up evaluations.

### Ethical Approval:

The study protocol was reviewed and approved by the Institutional Review Board (IRB No:2198; Dated October 17, 2024). Written informed consent was obtained from each patient.

Demographic data like age and gender and clinical characteristics like EE presence and severity (LA-A to D) and hiatus hernia (present/absent) were assessed and documented based on endoscopic findings at baseline. After this, all patients were given vonoprazan 20 mg orally once daily for eight weeks. No additional PPIs, H2-receptor antagonists, or prokinetic agents were allowed during this time. Participants were counseled about lifestyle modifications including weight management, avoidance of large/late meals and head-of-bed elevation during sleep. After completion of treatment, a second upper GI endoscopy was performed to re-evaluate erosive esophagitis presence and severity using the LA classification.

### Statistical analysis:

Data was entered into and analyzed using SPSS version 23. Continuous variables like age were reported as means with standard deviations. categorical variables were summarized as frequencies and percentages. The primary outcome was assessing healing of EE from baseline to post-treatment, while secondary outcome was effect of gender and hiatus hernia on treatment outcome. Statistical comparison of pre- and post-treatment esophagitis presence was done. A p-value of less than 0.05 was considered statistically significant. McNemar’s test was used for comparisons and matched-pair odds ratio with 95% CI was calculated. Association between post-treatment esophagitis presence and gender and hiatus hernia was established using logistic.

## RESULTS

The demographic and clinical characteristics of patients, as well as esophagitis (presence/absence and grades) before and after treatment are II summarized in Table-I and II. The mean age of the patients was 42.51±8.19 years. Regarding gender, 120(54.5%) were male out of 220 participants. Based on the initial endoscopic evaluation where all patients had erosive esophagitis, 115(52.3%) had Grade-C, and 26(11.8%) had Grade-D esophagitis. Following treatment, second endoscopy showed that 191(86.8%) had complete healing of esophagitis, while 29(13.2%) still had esophagitis out of which 18(8.2%) exhibited LA-A and 11(5%) had LA-B esophagitis (OR:6.59, 95%CI 4.45 - 9.73, p < 0.001).

**Table-I:** Patient’s Characteristics and Reflux Severity Before and After Treatment.

Variables				Mean±SD n(%)
Age (Years)				42.51±8.19
Gender			Male	120(54.5%)
			Female	100(45.5%)
Erosive Esophagitis at 1^st^ Endoscopy	*Present, n(%)*	*Absent, n(%)*	*Grades*	
	210 (100 %)	0(0%)	LA - B	79(35.9%)
			LA - C	115(52.3%)
			LA - D	26(11.8%)
Erosive Esophagitis at 2^nd^ Endoscopy	29(13.2%)	191(86.8%)	No Esophagitis	191(86.8%)
			LA - A	18(8.2%)
			LA - B	11(5%)
Hiatus Hernia			Present	58(26.4%)
			Not Present	162(73.6%)

**Table-II:** Comparison of reflux severity before and after treatment by gender and presence of hiatus hernia.

*Variable*			*Before Treatment, n(%)*	*After Treatment, n(%)*	*p-value* *OR, 95%CI*
Erosive Esophagitis (Gender)	Male (n=120)	No Esophagitis	0(0.0%)	104(86.6%)	>0.05 (0.94)
		LA - A	0(0.0%)	9(7.5%)	
		LA - B	44(36.66%)	7(5.8%)	
		LA - C	60(50.0%)	0(0.0%)	
		LA - D	16(13.33%)	0(0.0%)	
		Total(present/absent)	120(120/0)	120 (16/104)	
	Female (n=100)	No Esophagitis	0(0.0%)	87(87.0%)	
		LA - A	0(0.0%)	9(9.0%)	
		LA - B	35(35%)	4(4.0%)	
		LA - C	55(55%)	0(0.0%)	
		LA - D	10(10%)	0(0.0%)	
		Total(present/absent)	100(100/0)	100(13/87)	
Erosive Esophagitis (Hernia)	Hernia Present (n=58)	No Esophagitis	0(0.0%)	35(60.34%)	<0.001 OR:17.08 (6.47- 45.08)
		LA - A	0(0.0%)	14(24.13%)	
		LA - B	9(15.5%)	9(15.52%)	
		LA - C	29(50.0%)	0(0.0%)	
		LA - D	20(34-.40%)	0(0.0%)	
		Total(present/absent)	58 (58/0)	58 (23/35)	
	Hernia Absent (n=162)	No Esophagitis	0(0.0%)	156(96.29%)	
		LA - A	0(0.0%)	4(2.46%)	
		LA - B	70(43.2%)	2(1.24%)	
		LA - C	86(53.0%)	0(0.0%)	
		LA - D	6(03.7%)	0(0.0%)	
		Total(present/absent)	162(162/0)	162(6/156)	
Overall	Erosive Esophagitis (n=220)	No Esophagitis	0(0.0%)	191(86.8%)	<0.001 OR:6.59 (4.45 - 9.73)
		LA - A	0(0.0%)	18(8.2%)	
		LA - B	79(35.9%)	11(5%)	
		LA - C	115(52.3%)	0(0.0%)	
		LA - D	26(11.8%)	0(0.0%)	
		Total(present/absent)	220 (220/0)	220(29/191)	

In Table-I and II, presence/absence and grades of esophagitis are compared based on gender and hiatus hernia. Among 120 male patients, all had esophagitis before treatment with 60(50.0%) having as LA-C and 16(13.33%) having LA-D esophagitis, but following treatment, 104(86.6%) had no esophagitis on repeat endoscopy with only nine (7.5%) having LA-A and seven (5.8%) having LA - B esophagitis. Among 100 females, all had esophagitis before treatment with 55(55%) having LA-C and 10(10%) having LA-D esophagitis; however, post-treatment 87(87.0%) showed no esophagitis with only nine (9.0%) having LA-A and four (4%) having LA-B esophagitis. These results show no significant difference in healing rates between male and female patients (p=0.94).

Hiatus hernia was present in 58(26.4%) patients and none were initially free of esophagitis with 29(50.0%) having LA-C and 20(34-.40%) having LA-D esophagitis. After treatment 35(60.34%) had no esophagitis while 23(39.65%) still had esophagitis out of which 14(24.13%) had LA-A and nine (15.52%) had LA-B esophagitis. Among 162 individuals without hernia, all of whom had EE before treatment, 156(96.29%) were free of esophagitis following treatment (OR:17.08, CI 6.47 - 45.08, p < 0.001). Among these patients, initially 86 (53.0%) had LA-C and six (03.7%) had LA-D esophagitis which improved to four (2.46%) having LA-A and two (1.24%) with LA-B esophagitis after treatment.

**Fig.1:**
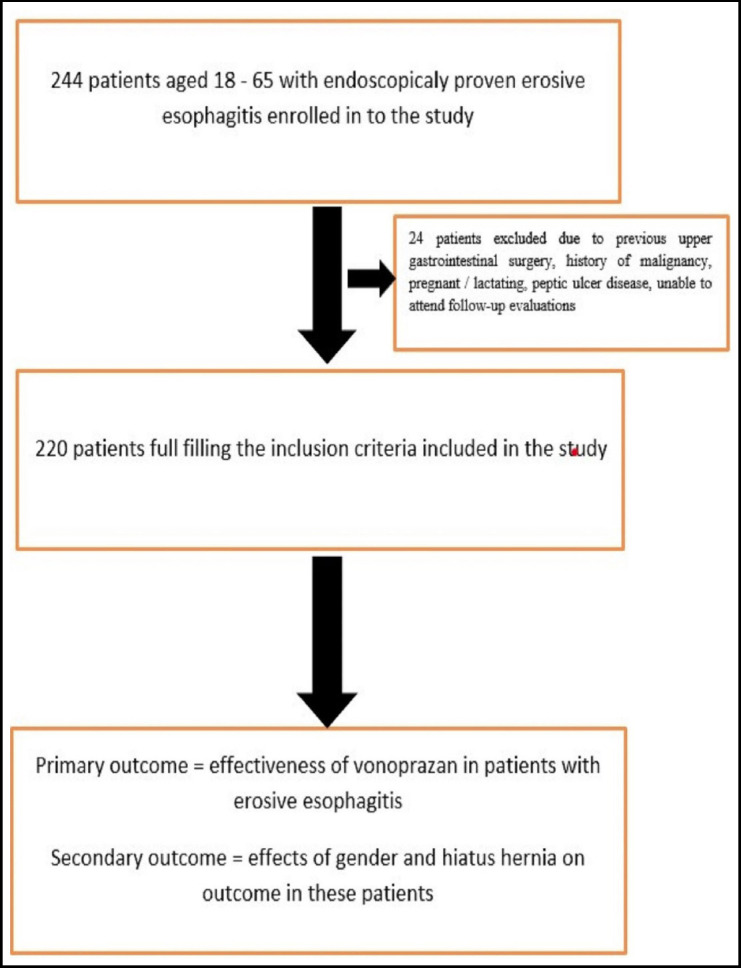
Study flow diagram.

## DISCUSSION

This study demonstrated a high mucosal healing rate (86.8%) in patients with erosive esophagitis (EE) treated with vonoprazan thus aligning with contemporary evidence supporting its efficacy in managing EE.

A prospective study by Hoshikawa et al. showed that 86.2% of patients with mild EE (LA-A/B) maintained remission on on-demand vonoprazan 20 mg, with 100% of grade A and 78.9% of Grade-B cases healed.^14^ In our study 64.1% patients had LA - C/D esophagitis and 86.8% patients showed complete healing (OR:6.59, 95%CI 4.45 - 9.73, p < 0.001) as shown in Table-I and II, reinforcing the role of vonoprazan for treatment of EE.

A RCT by Laine et al. involving EE patients compared vonoprazan 20 mg daily to lansoprazole 30 mg reported healing rates of 92.9 % for vonoprazan vs 84.6% for lansoprazole, particularly in LA-C/D esophagitis, with superior maintenance of healing at eight weeks.^1^ Our results with 86.8% complete mucosal healing rate, particularly in LA-C/D esophagitis support vonoprazan’s efficacy in severe EE cases with its applicability in routine clinical settings.

The VISION Trial(NCT02679508), involving 208 patients with EE, evaluated vonoprazan 10 mg reported healing rates exceeding 90% with minimal adverse events.^15^ Though our study spanned only eight-weeks, the overall healing rate of 86.8% with complete resolution of LA-C/D esophagitis suggests vonoprazan’s short-term effectiveness and potential for long-term therapeutic benefits. These outcomes are consistent with the VISION trial, underscoring vonoprazan’s rapid and sustained efficacy.

A multicenter study by Xiao Y et al. in Asian patients reported healing rates over 90% at Eight weeks with vonoprazan in patients with EE of varying severity with minimal adverse events.^16^ Our observed 86.8% healing rate, particularly in the LA-C/D subgroup, reflects similar high efficacy, reinforcing vonoprazan’s therapeutic benefits in routine practice.

The Saudi Gastroenterology Association consensus (2024) found vonoprazan non-inferior overall but superior in mucosal healing rates in severe cases (LA-C/D) of EE compared to lansoprazole.^17^ In our study, 86.8% patients had complete resolution of esophagitis after eight weeks treatment with vonoprazan with 64.1% patients having LA-C/D esophagitis before treatment which was completely absent after treatment showing excellent efficacy of vonoprazan in this group of patients, thus closely aligning with above mentioned findings. This reinforces vonoprazan’s clinical utility in EE and its inclusion in treatment guidelines for advanced EE.

A study by Nguyen et al. involving 862 patients undergoing hiatal hernia repair reported persistent symptoms in one-third of patients who did not receive surgical intervention, despite being on acid suppression therapy.^18^ Similarly, in our study, 60.34% (n=35) patients with hiatus hernia and EE had no esophagitis while 39.65%(n=23) still had esophagitis after eight weeks of vonoprazan (OR:17.08, CI 6.47 - 45.08, p < 0.001), indicating statistically significant association between presence/absence of hiatus hernia and healing rates of EE after treatment with vonoprazan. Various mechanisms may contribute to the adverse effect of hiatus hernia on the effectiveness of vonoprazan in erosive esophagitis. Some of them include displacement of gastroesophageal junction in to the thoracic cavity may impair the competence of lower esophageal sphincter esophageal clearance is compromised in the presence of hiatus hernia and reduced diaphragmatic support thus prolonging exposure of esophageal mucosa to gastric contents – hiatus hernia may create a gastric acid pocket above diaphragm which may serve as a reservoir for gastric acid postprandially thus reducing the effectiveness of acid suppressing medication large hiatus hernias are associated with prolonged acid exposure time and more sever reflux episode thus leading to delayed healing of mucosa even in the presence of potent acid suppressing medications like vonoprazan.

A study by Shahsavari et al. involving 1244 patients showed that larger hiatal hernias (≥2 cm) were linked with lower LES pressures, increased acid exposure time and more severe reflux episodes and thus increases risk of EE with reduced response to acid suppression therapy.^19^ Consistent with this, our study showed that 39.65% of patients having hiatus hernia had residual esophagitis despite eight weeks treatment with vonoprazan as compared to 96.29% complete resolution of esophagitis after treatment with vonoprazan in those without hiatus hernia, which is highly statistically significant (OR:17.08, CI 6.47 - 45.08, p < 0.001), highlighting impact of anatomical disruption on treatment efficacy, even with strong acid suppression.

Our study also assessed effects of gender on healing rate of esophagitis at the end of eight weeks treatment with vonoprazan, with no significant difference found between male and female patients in terms of healing rates i.e. 86.6% vs 87% complete resolution of esophagitis respectively (p=0.94) at the end of treatment duration, Table-II.

### Strengths of our study:

It is prospective design, use of Los Angeles Classification for grading erosive esophagitis which is a standardized grading system for it, adequate sample size, and evaluation of real-world data of effectiveness of vonoprazan in clinically relevant population.

### Limitations:

It include the use of a non-probability sampling technique that may limit generalizability, no assessment of symptom based outcomes, no subclassification of hiatus hernia which might influence treatment outcomes and non-inclusion various lifestyle variables like BMI, smoking etc. All highlights the need for further larger prospective trials.

## CONCLUSION

Vonoprazan demonstrated high efficacy in healing EE. Outcomes were equally effective in male and female patients while more effective in patients without a hiatus hernia. This highlight vonoprazan’s therapeutic potential in EE and the need to consider individual patient factors when managing EE.

### Authors’ Contribution:

**MSK:** Conceived, designed and did statistical analysis & editing of manuscript and is responsible for integrity of research. **MI, NJ & AR:** Did literature search, data collection and manuscript writing.**AR:** Critical review and final approval of manuscript.

## References

[1] Laine L, DeVault K, Katz P, Mitev S, Lowe J, Hunt B (2023). Vonoprazan Versus Lansoprazole for Healing and Maintenance of Healing of Erosive Esophagitis: A Randomized Trial. Gastroenterology.

[2] Chandan S, Deliwala S, Mohan BP, Ramai D, Dhindsa B, Bapaye J (2023). Vonoprazan versus lansoprazole in erosive esophagitis-A systematic review and meta-analysis of randomized controlled trials. Indian J Gastroenterol.

[3] Fung S (2025). Vonoprazan: A Review in Erosive Esophagitis and Non-Erosive Gastro-Esophageal Reflux Disease. Drugs.

[4] Simadibrata DM, Lesmana E, Fass R (2024). Vonoprazan is superior to lansoprazole for healing of severe but not mild erosive esophagitis: a systematic review with meta-analysis of randomized controlled trials. J Gastroenterol Hepatol.

[5] Xiao Y, Qian J, Zhang S, Dai N, Chun HJ, Chiu C (2024). Vonoprazan 10 mg or 20 mg vs. lansoprazole 15 mg as maintenance therapy in Asian patients with healed erosive esophagitis: A randomized controlled trial. Chin Med J (Engl).

[6] Uemura N, Kinoshita Y, Haruma K, Kushima R, Yao T, Akiyama J (2025). Vonoprazan as a long-term maintenance treatment for erosive esophagitis: VISION, a 5-year, randomized, open-label study. Clin Gastroenterol Hepatol.

[7] Kothadia JP, Howden CW (2024). Potassium-competitive acid blockers for the treatment of gastroesophageal reflux disease. Foregut.

[8] Van Zanten SV (2024). In Grade-C/D erosive esophagitis, vonoprazan ranks highest among PPIs and P-CABs for healing and maintaining remission. Ann Intern Med.

[9] Frazzoni L, Fuccio L, Zagari RM (2023). Management of gastro-esophageal reflux disease: Practice-oriented answers to clinical questions. World J Gastroenterol.

[10] Ashida K, Iwakiri K, Hiramatsu N, Sakurai Y, Hori T, Kudou K (2018). Maintenance for healed erosive esophagitis: phase III comparison of vonoprazan with lansoprazole. World J Gastroenterol.

[11] Okanobu H, Kohno T, Mouri R, Hatsushika Y, Yamashita Y, Miyaki E (2021). Efficacy of vonoprazan 10 mg compared with 20 mg for the initial treatment in patients with erosive esophagitis: a randomized pilot study. Esophagus.

[12] Xiao Y, Wang Q, Li G, Nail A, Song Q, Xie L (2023). PCR5 First Interim Analysis of Patient-Reported Outcomes in View Study: A Multicenter, Single-Arm, Prospective, Non-Interventional Study of Vonoprazan in Real-World Clinical Practice in China. Value Heal.

[13] Tutuian G, Leandri C, Tutuian R, Scialom S, Leconte M, Dohan A (2023). Achalasia and hiatal hernia: a rare association and a therapeutic challenge. J Neurogastroenterol Motil.

[14] Hoshikawa Y, Koeda M, Rokugo T, Momma E, Kawami N, Iwakiri K (2025). Long-term efficacy of on-demand vonoprazan treatment for mild reflux esophagitis: success rates and predictors of treatment failure. Esophagus [Internet].

[15] Haruma K, Kinoshita Y, Yao T, Kushima R, Akiyama J, Aoyama N (2023). Randomised clinical trial: 3-year interim analysis results of the VISION trial to evaluate the long-term safety of vonoprazan as maintenance treatment in patients with erosive oesophagitis. BMC Gastroenterol.

[16] Xiao Y, Zhang S, Dai N, Fei G, Goh KL, Chun HJ (2020). Phase III, randomised, double-blind, multicentre study to evaluate the efficacy and safety of vonoprazan compared with lansoprazole in Asian patients with erosive oesophagitis. Gut.

[17] Alzahrani MA, Alqaraawi AM, Alzubide SR, Abufarhaneh E, Alkhowaiter SS, Alsulaimi M (2024). The Saudi Gastroenterology Association consensus on the clinical care pathway for the diagnosis and treatment of GERD. Saudi J Gastroenterol.

[18] Nguyen CL, Tovmassian D, Isaacs A, Gooley S, Falk GL (2023). Trends in outcomes of 862 giant hiatus hernia repairs over 30 years. Hernia.

[19] Shahsavari D, Smith MS, Malik Z, Parkman HP (2021). S471 Hiatal Hernias Associated with Acid Reflux: Size Larger Than 2 cm Matters. Off J Am Coll Gastroenterol |ACG.

